# Preliminary results of a multidimensional approach to screen for frailty in community-dwelling older adults of eight Italian regions: the SUNFRAIL+ study

**DOI:** 10.3389/fpubh.2025.1543724

**Published:** 2025-04-15

**Authors:** Vincenzo De Luca, Clara Donnoli, Valeria Formosa, Edoardo Carnevale, Michele Bisogno, Lola Patumi, Lisa Leonardini, Paola Obbia, Ernesto Palummeri, Manuela Ruatta, Anna Maddalena Basso, Marcello Minichini, Daniela Adamo, Dario Bruzzese, Lorenzo Mercurio, Michele Virgolesi, Stefano Toccoli, Simona Sforzin, Fortunata Denisi, Moira Borgioli, Marino Dell’Acqua, Valentina Cacciapuoti, Guido Iaccarino, Giuseppe Liotta, Maddalena Illario

**Affiliations:** ^1^Department of Public Health, Federico II University of Naples, Naples, Italy; ^2^Department of Biomedicine and Prevention, Tor Vergata University of Rome, Rome, Italy; ^3^Programma Mattone Internazionale Salute, ULSS 4 Veneto Orientale Local Health Agency, San Donà di Piave, Italy; ^4^Directorate of Health Professions, Local Health Agency Cuneo 1, Cuneo, Italy; ^5^Ligurian Health Agency, Liguria Region, Genoa, Italy; ^6^Department of Primary Care and District Activities, Ligurian Social Health Agency 4, Genoa, Italy; ^7^Department of Neuroscience, Reproductive Sciences and Dentistry, Federico II University of Naples, Naples, Italy; ^8^Department of Primary Care, Provincial Health Authority of Trento, Trento, Italy; ^9^Social Cooperative “Res Omnia”, Reggio Calabria, Italy; ^10^Design, Development, Training and Research Unit, North-West Local Health Authority, Pisa, Italy; ^11^Socio-Health Department, Insubria Health Protection Agency, Varese, Italy; ^12^Socio-Health Department, Valle Olona Territorial Socio-Health Agency, Varese, Italy; ^13^Department of Clinical Medicine and Surgery, Federico II University of Naples, Naples, Italy

**Keywords:** frailty, screening, aging, bio-psycho-social domains, multidimensional assessment, community-dwelling older adults, prevention, health promotion

## Abstract

**Background:**

Frailty syndrome in older adults is an age-related decline in many physiological systems, that results in a reduced response to minor stressors, and leading to an increased risk of falls, hospitalization, disability and death. Frailty can be prevented, reversed or mitigated by early screening for frailty risk in community-dwelling older adults, allowing for preventive interventions on clinical and social determinants.

**Objectives:**

The present article reports the preliminary results of the SUNFRAIL+ study multidimensional cascade assessment in a group of community-dwelling older adults of 8 Italian regions aimed to stratify the population according to the needs of care at the first administration, integrated with the quality of life (QoL) assessment to evaluate the impact of early, integrated care.

**Methods:**

The SUNFRAIL+ study performed a multidimensional assessment of community-dwelling older adults by linking elements of the SUNFRAIL frailty assessment tool with an in-depth assessment of biopsychosocial domains of frailty, through validated questionnaires and physical tests.

**Results:**

The sample consisted of 743 participants (279 males and 464 females). The mean score of the multidimensional assessment with the SUNFRAIL tool was 2.31 (SD ± 0.7) with the cut-off point of frailty set at 3. The analysis revealed a significant difference in SUNFRAIL scores between the age groups. The results confirmed that individuals with higher frailty scores are significantly more likely to experience falls. Significant and conceptually valid correlations were found between physical and cognitive tests and QoL scores. Frailty is significantly associated with a lower physical and mental quality of life. The results indicated that older age and lower education levels are associated with higher frailty scores.

**Conclusion:**

The study demonstrates important different correlation levels, firstly between the assessment of frailty by SUNFRAIL and the perceived QoL; secondarily among all SUNFRAIL spheres and their second test sets that more objectively evaluate each frailty domain. The study demonstrates that the SUNFRAIL+ approach accurately assesses frailty status owing to its strong correlation with the SF-12 quality of life assessment.

## 1 Introduction

Frailty syndrome in older adults is an age-related decline in many physiological systems, resulting in a reduced response to minor stressors, and leading to an increased risk of falls, hospitalization, disability and death (1). Due to demographic changes and the progressive aging of the global population with the consequent increasing demand for care services and complexity of health needs of the population over 65, frailty represents a serious challenge for public health and a growing economic burden on health systems (2). Frailty leads to a deterioration in the quality of life of older adults, affecting their social habits and access to health and social services, resulting in an increasing commitment to their closest relatives (3). Recent evidence confirms that the overall prevalence of frailty in community-dwelling older adults ranges from 11% among those who were 50 to 59 years of age to 51% among those who were 90 years (4).

There is no single clinical definition of frailty that is universally accepted and translated into clinical practice. Frailty is defined within two main paradigms: biomedical and bio-psycho-social. According to the biomedical paradigm (5), frailty is characterized by a reduction in functional reserves and resistance to stressors due to a cumulative decline of physiological systems causing vulnerability and adverse events. The bio-psycho-social paradigm (6) defines frailty as a dynamic state that affects individuals with losses in one or more functional areas (physical, psychological and social), overall increasing the risk of adverse outcomes. Within the bio-psycho-social approach, several factors are taken into consideration, such as medical, environmental, educational, economic and psychological aspects, which overall require a more holistic point of view of the patient and his difficult situation.

The health needs of older adults increase based on the degree of frailty and are accompanied by a loss of Activities of Daily Living (ADL) and an increase in pharmacotherapy, posing more risk of adverse health outcomes (2).

Frailty and its consequences can be prevented, reversed or mitigated by personalized interventions (7). Key interventions to improve health outcomes for individuals who are frail or at risk of frailty include exercise, nutrition, multicomponent interventions, psychosocial or cognitive training, home telemonitoring and personalized geriatric care models (8, 9). The role of lifestyles, therefore of primary prevention, in determining frailty is indispensable, just as tertiary prevention plays a very important interaction in the management of the different moments of frailty, in reference to chronic conditions. The sustainability of interventions to contrast frailty depends on the severity of frailty, but above all on the ability to engage the patient in changes in their lifestyle with respect to their health conditions. In older adults at risk of frailty, the occurrence of an acute event, with consequent institutionalization and hospitalization, can lead to a rapid worsening of the patient’s health conditions in all areas of frailty, with lower chances of recovery (10, 11).

Early diagnosis of frailty is essential to prevent or delay disabilities in older adults living at home (12). For this reason, the European consensus group, ADVANTAGE, has recommended screening all over 70s, in all encounters with healthcare professionals (13). Early screening for frailty risk in community-dwelling older adults allows for preventive intervention on the clinical and social determinants of frailty and thus the prevention of adverse events (14). The optimal timing and the most suitable tool for screening for frailty are a matter of debate. Most screening and assessment tools for the early detection of functional deficits are coded to distinguish between frailty and disability or are targeted to a single dimension/domain (15). Tools such as the Frailty Phenotype (16) and the Frailty Scale (17), are more focused on the analysis of the physical domain, require a lot of time and are scarcely used in daily practice, especially in primary care settings (18). In order to implement interventions capable of slowing down the progression toward disability, the assessment of frailty requires a comprehensive analysis of physical-functional, socio-environmental-economic, educational and psychological contributions (19). In daily clinical practice, frailty screening has implemented in outpatient clinics and hospitals through a comprehensive geriatric assessment (CGA) to allow for individualized and person-centered interventions (20).

The “Reference Sites Network for Prevention and Care of Frailty and Chronic Conditions in community dwelling persons of EU Countries”—SUNFRAIL project, funded by the European Commission, under the Third Health Programme (grant no. 664291), involved multiple stakeholders to identify innovative approaches to frailty in community-dwelling older adults, in order to create standardized interventions that can take into account all the different factors that influence the individual’s health status toward frailty. The model identified by the SUNFRAIL consortium considers it important to evaluate the risk factors of frailty, to prevent it and maintain the patient’s independence. In the SUNFRAIL model, frailty and its risk factors can be identified in community health, social and informal systems, by appropriately trained professionals and carers, who can activate an initial “alarm” for further prevention activities, specialist investigations and diagnoses (21). Based on this approach, the SUNFRAIL consortium has developed and validated a tool for the early identification of frailty in the over-65 population in different settings, which allows to generate alerts that guide subsequent diagnostic assessments for health promotion, disease prevention and targeted interventions (22). The screening tool, which consists of only nine items, can be used by general practitioners or other health service professionals and community actors, who can link specific items to other in-depth tools for the assessment of specific dimensions. The working group on frailty of the International Health Brick Program (ProMIS) of the Italian Ministry of Health (23) has linked the elements of the SUNFRAIL tool to additional scales aimed at assessing the domains of frailty, developing a new service model for the screening of frailty in community-dwelling older adults, confirming or not the presence of risk factors according to a biopsychosocial approach, such as: adherence to prescription and polypharmacy; nutrition; physical activity; adherence to medical visits; falls; cognitive decline; loneliness; support network; and socioeconomic conditions (24). The present article reports the preliminary results of the SUNFRAIL+ study multidimensional cascade assessment, aimed to stratify the population according to the needs of care at the first administration, integrated with the Quality of Life assessment of community-dwelling older adults in 8 Italian regions.

## 2 Methods

Another publication has described the study protocol in detail (25).

### 2.1 Study design, population and settings

This cross-sectional study was conducted under the International Health Brick Program (ProMIS) of the Italian Ministry of Health, whose mission is to promote interchange and collaboration between Italian, European, and non-European health systems.

Organizations responsible for recruiting the sample were: the Department of Public Health of the University of Naples Federico II (Campania Region), the Socio-Health Authority Ligure no. 4 (Liguria Region), the Trento Provincial Socio-Health Authority (Autonomous Province of Trento), the Social Cooperative “Res Omnia” (Calabria Region), the Cuneo 1 Local Health Authority (Piemonte Region), The Northwest Local Health Unit (Tuscany Region), Territorial Health and Social Authority of the Olona Valley (Lombardy Region) and the Department of Biomedicine and Prevention of the University of Rome Tor Vergata (Lazio Region).

Exclusion criteria were:

(1) People aged under 65
(2) Residents in assisted-living facilities or nursing homes
(3) Being unable to understand the questionnaires or sign the informed consent.

The methodology used to select individuals was non-probability sampling. The sample consisted of males and females over 65, living at home and independently recruited from each center after signing informed consent. Each center recruited at least 100 individuals.

### 2.2 Measurements

#### 2.2.1 SUNFRAIL tool and SUNFRAIL+

SUNFRAIL comprises 9 items that investigate biopsychosocial frailty across three domains: physical, psychological and socio-economic, with five, one, and three items, respectively (22, 26). Each item generates one point if the alert is triggered. The maximum total score that can be achieved is 9. The higher the score, the alert for frailty (26) (Table 1).

**Table 1:** The SUNFRAIL checklist (22).

Questions		
1. Do you regularly take 5 or more medications per day?	Yes (Alert)	No
2. Have you recently lost weight such that your clothing has become looser?	Yes (Alert)	No
3. Your physical state made you walking less during the last year?	Yes (Alert)	No
4. Have you been evaluated by your GP during the last year?	Yes	No (Alert)
5. Have you fallen 1 or more times during the last year?	Yes (Alert)	No
6. Have you experienced memory decline during the last year?	Yes (Alert)	No
7. Do you feel lonely most of the time?	Yes (Alert)	No
8. In case of need, can you count on someone close to you?	Yes	No (Alert)
9. Have you had any financial difficulties in facing dental care and health care costs during the last year?	Yes (Alert)	No

When the item generates an alert, other specific validated instruments are triggered for further evaluation. Table 2 summarizes all instruments in the SUNFRAIL+ tool including their domain origin, goal, parameters and cut-off scores. The secondary tools are already included in the SUNFRAIL+ platform (25). The instruments were chosen by a panel of experts as mentioned in the previous published protocol. The item regarding prescription adherence included a questionnaire (MARS) that was later changed to TAS (Therapeutic Adherence Scale), consisting of 4 items to measure medication adherence (27).

**Table 2:** Second-level scales were administered in case of positive alerts to SUNFRAIL+ items.

Secondary tool	Corresponding SUNFRAIL item	Description	Total score
TAS (Therapeutic Adherence Scale) (27)	1	The test is composed of 4 items investigating adherence (scoring from 0 to 1)	0–2: nonadherent 3–4: adherent
PREDIMED (Assessment of Adherence to the Mediterranean Diet) (48)	2	The test is composed of 14 items measuring adherence to the Mediterranean diet (scoring from 0 to 1)	≤5: poor adherence 6–9: medium adherence ≥10: good adherence
MNA (Mini-Nutritional Assessment) (49)	2	The test is composed of 18 items identifying the older adults at risk of malnutrition allowing for early intervention	24–30: normal nutritional status 17–23.5: risk of malnutrition less than 17: poor nutritional status
SPPB (Short Physical Performance Battery) (50)	3	The test is composed of 3 sections evaluating balance, walking and sit to stand; scores from 1 to 4	From 0 (worst performance) to 12 (best performance)
General Practitioner (GP) visiting checklist (25)	4	The test is composed of 7 questions assessing adherence to medical visits	The questionnaire does not have a score but is positive if the person is non-adherent to at least one item
AFEAT (Aged-friendly environment assessment tool) (51)	5	The test is composed of 10 questions scored on a 5-point Likert scale, measuring how friendly the living and community environment is perceived	From 10 (low age-friendly environment) to 50 (high age-friendly environment)
TUG (Timed Up and Go) Test (52)	5	The test is used to assess mobility and consists of getting up from a chair, walking a 3-meter distance, going back, and sitting down	The less time the person takes, the better the performance
QMCI (Quick Mild Cognitive Impairment) (53)	6	Cognitive screening tool to assess cognitive function (orientation, registration, clock drawing, delayed recall, verbal fluency and logical memory)	The Equivalent score of 0 in the Italian validation study is ≤49.4
GPCOG (General practitioner assessment of cognition) (54)	6	The test measures cognitive function and is usable in general practice. It consists of 2 parts: the evaluation of the patient and the interview with the family/informant	First part score of 9: normal First part score of 5–8 plus second part score ≤ 3: cognitive deficit First part score of 5–8 plus second part score 4–6: MCI, to be monitored over time
GDS (Geriatric Depression Scale) (55)	7	The test (short version, 15 items) detects the presence of depressive symptoms in the older person	≤ 5: depression unlikely 6–9: possible depression ≥10: probable depression
SPS (Social Provision Scale) (56)	8	The test is the short version (10 items) and each question is scored on a Likert scale from 1 to 4. It measures the level of social support available	The higher the final score the stronger is the social support
MUSE (socio-economic conditions self-assessment questionnaire) (57)	9	It is composed of 9 socio-economic variables	There is no final score

#### 2.2.2 Quality of life: the SF-12 health survey

Quality of Life is assessed by SF-12 (Short-Form Health Survey) v.1 (28), the short form of the SF-36 (29). This tool has been validated in Italian (30) and consists of 2 parts that measure perceived physical and mental health. It analyses 8 domains: physical functioning, role limitations due to physical health problems, role limitations due to emotional health problems, mental health, body pain, general health, vitality, and social functioning (30). It can be administered in a few minutes, and its final score is obtained using an algorithm. The higher the final score, the better the perceived physical and mental health.

### 2.3 Data collection

Data was collected from February 2023 to May 2024. After signing the informed consent, sex, age, education, and geographical area were recorded during the user interview. The SUNFRAIL tool (22) was administered to assess multidimensional frailty in older adults. Subsequently, if a SUNFRAIL item alert has been triggered, an in-depth evaluation based on the identified risk domain through specific validated scales was performed (Table 2). To conclude, Quality of Life (QoL) was assessed by Short Form-12 (SF-12) v.1. An ICP (Individualized Care Plan), was filled in, based on the summary of all the physical, psychological, and socio-economic characteristics examined, and the suggested intervention was also entered into the digital platform. Depending on the positive alerts from SUNFRAIL+, each center implemented prevention and health promotion interventions to prevent the onset or worsening of frailty. Thus, interventions were carried out to improve empowerment and adherence to medical appointments, as well as nutritional education, fall prevention, and socialization activities. Periodical visits were scheduled to follow up on the impact of the ICPs as well as the evolution of frailty.

### 2.4 Ethics statement

The research protocol was registered at ClinicalTrials.gov on 9 December 2022 (registration number: NCT05646472) and was approved by the local Ethics Committee of University “Federico II” - Azienda Ospedaliera di Rilievo Nazionale “A. Cardarelli” (N. 284/22) as lead partner. Each center has submitted the protocol to its local committee for revision and approval.

All participants signed the information notice, the informed consent and the data processing consent. Data were pseudo-anonymized by assigning to each user a unique code. The online dataset was password-protected.

### 2.5 Statistical analysis

The complete questionnaires have been analyzed, focusing on different aspects. Preliminary, descriptive and confirmatory analyses were conducted, such as bivariate and multivariate analyses. The main statistical tests used were parametric and non-parametric. The Normality was tested using the Shapiro–Wilk test. For normally distributed variables the comparisons between the means of two groups were performed using *t*-test, whereas for non-normally distributed variables, the Mann–Whitney U test was applied. It has been applied also One-way ANOVA (Kruskal-Wallis test) for the comparison among more than two non-normally distributed variables. Correlation analyses were performed using Spearman’s correlation (non-normally distributed variables) among all secondary instruments for the questionnaires. Finally, it has been performed Logistic Regression and Generalized Linear Models. The Logistic Regression model included, as covariates and predictors of frailty scores, age, educational level, gender, and geographical area, while as dependent variable the SUNFRAIL level of frailty. The two GLMs have been introduced as dependent variables, respectively, the SF-12 PCS (Physical Component Summary) and MCS (Mental Component Summery), and as covariates age, gender, the number of positive alerts by SUNFRAIL, geographical area and education level.

IBM SPSS Statistics software v.26 and Jamovi v. 2.3.21 were used for data processing, with *p*-values <0.05 considered statistically significant. As explained in the already published Protocol, the number of people recruited in the study (743) was higher than the initially planned 195 (25), which allowed statistical analyses to be conducted with a lower probability of random error.

## 3 Results

### 3.1 Overview and participants characteristics

The sample was made up of 743 participants (279 males and 464 females) who gave their consent to participate to the study. Most of the sample was enrolled among the population living in the center of Italy (47.8%) while the remaining participants lived in the North and South regions (31.9 and 20.3% respectively).

Males accounted for 37.6% of the study participants. SUNFRAIL classified education into 3 levels: the lower with no school certificates, primary or middle school completion (56.9%), the middle included high school diploma (29.1%), and the higher one included bachelor, master or doctorate degree (14.1%). By classifying age into three categories, the sample was distributed as follows: 34.7% aged under 75, 43.8% aged 75–84, 21.6% over 85 with a mean age of 78.1 (SD ± 7.3; Table 3).

**Table 3:** Characteristics of the sample (*n* = 743).

	n (%)*
Age (*n* = 681)	
<75	236 (34.7%)
75–84	298 (43.8%)
>85	147 (21.6%)
Education level (*n* = 733)	
Low	417 (56.9%)
Medium	213 (29.1%)
High	103 (14.1%)
Sex (*n* = 743)	
Males	279 (37.6%)
Geographical distribution (*n* = 743)	
Northern Italy	237 (31.9%)
Central Italy	355 (47.8%)
Southern Italy	151 (20.3%)

*Percentages were calculated with reference to known values and excluding missing values.

### 3.2 SUNFRAIL questionnaire, the assessment of frailty and quality of life

All recruited individuals completed the SUNFRAIL tool questionnaire. The average score was 2.31 (SD ± 0.7) with the frailty cut-off point placed at 3 as explained by Gobbens et al. (26). The physical domain (items 1,2,3,4,5) presents the majority of active alerts compared to the psychological (6) and socio-economic (7, 8) domains as shown in Table 4. Among all people interviewed, 39.8% (*n* = 296, 101 males and 195 females) were found to be frail (total score ≥ 3). By stratifying frailty prevalence by age (under 75, 75–85 and over 85) the population is distributed as follows: 58 (8.5%) under 75, 124 (18.2%) aged 75–85 and 76 (11.2%) over 85 frails.

**Table 4:** Answers to SUNFRAIL items.

SUNFRAIL domains	SUNFRAIL item	Persons whose response generated an alert (n, %)
Physical domain	1. Do you regularly take 5 or more medications per day?	322 (43.3%)
	2. Have you recently lost weight such that your clothing has become looser?	109 (14.7%)
	3. Your physical state made you walking less during the last year?	311 (41.9%)
	4. Have you been evaluated by your GP during the last year?	154 (20.7%)
	5. Have you fallen 1 or more times during the last year?	175 (23.6%)
Psychological domain	6. Have you experienced memory decline during the last year?	280 (37.7%)
Socio-economic domain	7. Do you feel lonely most of the time?	194 (26.1%)
	8. In case of need, can you count on someone close to you?	62 (8.3%)
	9. Have you had any financial difficulties in facing dental care and health care costs during the last year?	111 (14.9%)

There was no missing data.

Regarding SF-12, the mean value in the entire sample was 43.5 (SD ± 9.9) for Physical Component Summary (PCS) and 49.5 (SD ± 9.7) for Mental Component Summary (MCS), resulting in a higher perceived mental health than perceived physical health. As fa as concern the gender, for males, the mean PCS value was 43.9 (SD ± 10.3) and the MCS value was 51.1 (SD ± 8.8), while for females the mean PCS was 43.4 (SD ± 9.7) and the MCS was 48.5 (SD ± 10.1). These scores showed a significant difference only in the MCS (*p* < 0.05).

#### 3.2.1 Bivariate analyses

The analysis among the differences in the SUNFRAIL domains and the total SUNFRAIL scores across different age groups revealed a significant difference in SUNFRAIL scores among the age groups (χ^2^ = 55.09, *p* < 0.001).

The analysis compared the level of frailty, as defined by the total SUNFRAIL score, with the incidence of falls (SUNFRAIL no.5), whether participants had fallen or not showed high statistical significance (*p* < 0.001), confirming that individuals with higher frailty scores are significantly more likely to experience falls.

A series of bivariate analyses were conducted to assess the correlations between the scores of several secondary tests (TAS, Therapeutic Adherence Scale; PREDIMED, Assessment of Adherence to the Mediterranean Diet; MNA, Mini-Nutritional Assessment; SPPB, Short Physical Performance Battery; GP, General Practitioner visiting checklist; AFEAT, Aged-friendly environment assessment tool; TUG, Timed Up and Go Test; QMCI, Quick Mild Cognitive Impairment; GPCOG, General practitioner assessment of cognition; GDS, Geriatric Depression Scale; SPS, Social Provision Scale; MUSE, Socio-economic conditions self-assessment questionnaire; and SF-12 PCS and MCS). In this case, the sample size was reduced to 503 participants due to missing data. The correlation matrix revealed numerous significant and conceptually valid correlations among these tests and quality of life (QoL) scores. Notably, tests related to physical domains, such as the Short Physical Performance Battery (SPPB) and the Timed Up and Go (TUG) test, showed significant correlations with the SF-12 physical score (PCS). Specifically, Spearman’s rank correlation coefficient for the SPPB was r = 0.354 (*p* < 0.001), indicating that higher SPPB scores, which reflect better physical performance, are associated with improved physical quality of life. Conversely, the TUG test showed a negative correlation (*r* = −0.316, *p* < 0.001), suggesting that higher TUG scores, which indicate increased fall risk, are associated with a lower physical quality of life.

**Figure 1:**
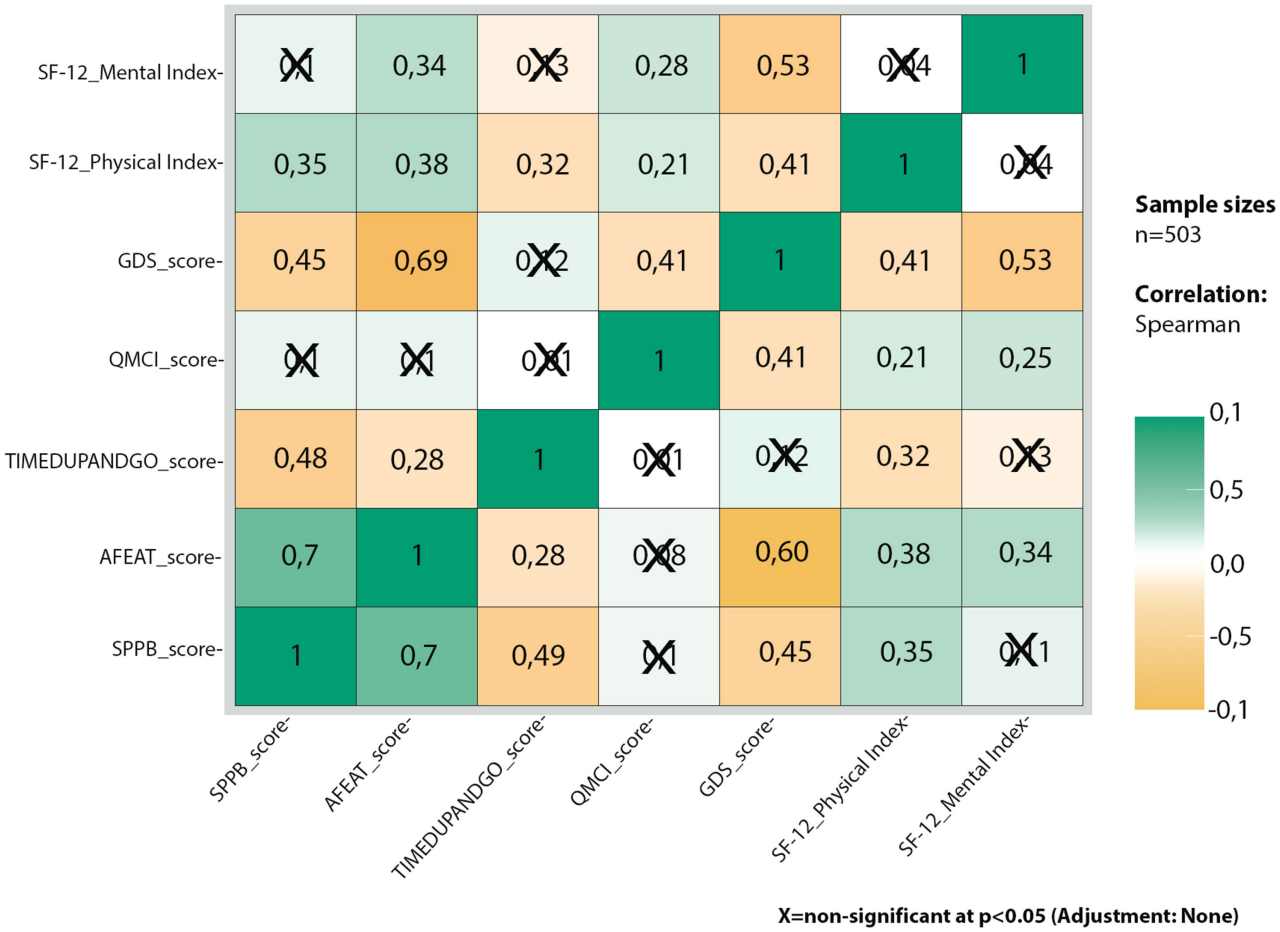
Correlation matrix among secondary tests set and SF-12 indices.

Similarly, tests related to the mental domain also showed significant correlations. The Geriatric Depression Scale (GDS) had a negative correlation with the SF-12 mental score (MCS; *r* = −0.534, *p* < 0.001), indicating that higher GDS scores, which suggest a greater likelihood of depression, are associated with lower mental quality of life. On the other hand, the Quick Mild Cognitive Impairment (QMCI) test showed a positive correlation (*r* = 0.257, *p* < 0.001), indicating that higher QMCI scores, reflecting better cognitive function, are associated with improved mental quality of life. These findings highlight the profound impact of both physical and mental health on the overall quality of life among older adults (Figure 1).

The differences in frailty status concerning physical and mental health indices established by the SF-12 revealed a significant difference between individuals with frail and non-frail scores in both the SF-12 physical and mental scores (*p* < 0.001). This suggests that frailty is significantly associated with lower physical and mental quality of life, highlighting the need for comprehensive assessment and intervention strategies to improve the well-being of frail individuals.

#### 3.2.2 Multivariate analyses

The logistic regression model indicated that both age and educational level significantly predicted the level of frailty, with older age and lower educational attainment being associated with higher frailty scores. Similarly, in the GLMs all variables emerged as significant predictors except in the case of geographical area for the GLM with MCS as the dependent variable and gender for GLM with PCS as the dependent variable (Table 5).

**Table 5:** Multivariate analysis.

Binomial logistic regression predicting frailty				
Variables	Estimates (ϐ)	Standard error	z-value	*p* value
Age (3 categories)	0.68	0.12	5.63	<0.001
Education Level (3 categories)	−0.28	0.12	−2.26	0.02
Gender (male = 0, female = 1)	0.23	0.17	1.72	0.09
Geographical area				
North-Center	0.41	0.19	2.12	0.03
South-Center	0.76	0.22	3.41	<0.001

Variables	Estimates (ϐ)	Standard error	z-value	*p* value	95% confidence interval	
					Lower	Upper
Generalized linear model (Dependent variable: physical component summary)						
Age	−0.16	0.06	−2.69	0.007	−0.28	−0.04
Number of positive alerts (SUNFRAIL tool)	−2.07	0.26	−7.87	<0.001	−2.59	−1.55
Geographical area (North-Center-South)	−1.61	0.59	−2.73	0.007	−2.77	−0.45
Education Level (3 categories)	2.23	0.57	3.92	<0.001	1.11	3.35
Generalized linear model (Dependent variable: mental component summary)						
Age	0.14	0.06	2.20	0.03	0.01	0.26
Number of positive alerts (SUNFRAIL tool)	−2.07	0.28	−7.63	<0.001	−2.60	−1.53
Geographical area (North-Center-South)	−0.35	0.61	−0.58	0.56	−1.54	0.84
Education Level (3 categories)	1.66	0.57	2.82	0.005	0.51	2.81

## 4 Discussion

The conducted research presents a comprehensive study on frailty assessment in community-dwelling older adults across multiple Italian regions. The SUNFRAIL+ study utilized a multidimensional cascade assessment approach to evaluate the impact of early, integrated care on the quality of life of older individuals. Indeed, the study demonstrates important different correlation levels, firstly between the assessment of frailty by SUNFRAIL and the perceived Quality of Life; secondarily among all SUNFRAIL spheres and their second test sets that more objectively evaluate each frailty domains (physical, mental and socio-economic).

The research demonstrated that frailty is significantly linked to lower physical and mental quality of life, emphasizing the need for comprehensive assessment and intervention strategies. This finding is particularly important as it highlights the far-reaching consequences of frailty beyond physical health, affecting an individual’s overall well-being and life satisfaction. The World Health Organization (WHO) defines Quality of Life as “individuals’ perceptions of their position in life in the context of the culture and value systems in which they live and in relation to their goals, expectations, standards and concerns” (31). A 2016 meta-analysis of 4 studies showed an association between frailty measured with the Fried Phenotype and Quality of Life measured with the Short Form Health Survey (32). Our research assessed frailty with a multidimensional instrument and shows that bio-psycho-social frailty is also associated with low quality of life. Preventive interventions aimed at counteracting the development of frailty can therefore also improve Quality of Life. Our study shows that, as has already been done in other countries (33), it would be useful in Italy to design and implement interventions to act on frail older adults, to improve not only their physical well-being but also their mental one. We are able to state that SUNFRAIL+ tool evaluates properly the state of frailty precisely due to the strong correlation with SF-12 Quality of Life assessment. The research also highlighted how depression and cognitive problems affect the quality of life of the older Italian people, hence the importance of also carrying out screening tests aimed at identifying these aspects in older persons. Depression (34) and cognitive deficits (35) are also associated with the development of frailty themselves.

This approach allowed researchers to capture a more nuanced understanding of frailty, considering various factors that contribute to an individual’s overall health and well-being. The study’s findings highlight the significant correlation among frailty, age, educational level, with older age and lower educational attainment associated with higher frailty scores. This correlation underscores the complex interplay between socioeconomic factors and health outcomes in older populations. The study shows that individuals with higher levels of education tended to have lower frailty scores, suggesting that cognitive engagement and lifelong learning may play a protective role against frailty. This confirms results obtained by other European research. For instance, a 2017 Netherlands study of 26,014 over-55 s showed that people with a low level of education had higher frailty scores, measured by the Frailty Index (36). Few studies have been conducted in Italy on the association between frailty and socio-economic status, so our study is important to confirm the association between education and frailty in our country as well, to raise awareness among stakeholders and policymakers on the importance of investing in equity.

Our study also demonstrated an association between frailty and the incidence of falls. The identification of frail older adults at risk of falling can therefore enable the implementation of interventions such as the exercise, which have shown effectiveness in preventing falls (37). Preventing falls in turn is of fundamental importance to reduce the incidence of disability and institutionalization (38). The SUNFRAIL+ study’s methodology included a range of assessments, such as grip strength measurements, gait speed tests and cognitive evaluations. This comprehensive approach enabled researchers to identify subtle signs of frailty that might be missed in more limited assessments, potentially allowing for earlier interventions and preventive measures, which could determine favorable effects on improving frailty or preventing its onset. In particular, the identification of adherence problems to therapy could direct the clinician toward deprescribing (39), or suggesting the adoption of IT tools, such as smart pill boxes or mobile app, that can facilitate the correct intake of drugs (40). The identification of an incorrect diet or malnutrition could direct the clinician toward a specific dietary regime or the use of particular nutritional supplements (41). The identification of motor problems could instead direct the clinician toward personalized exercises (42) or toward adapted physical activity, while the identification of cognitive or depressive problems could direct the clinician toward a specific therapy, even non-pharmacological (43, 44). Even the presence of socio-economic problems such as social isolation (45) or low income could determine the activation of a series of economic support measures (46) or the possible creation of a social support network.

In this context, it is interesting to mention the experience carried out by the Reference Site “Roma Tor Vergata,” part of Reference Site Collaborative Network (RSCN) with the “Prevention Days for Healthy and Active Ageing,” which are events that took place within the project “Long Live the Older adults!” of the Community of Sant’ Egidio. During these days, SUNFRAIL+ was administered to community-dwelling older adults and a personalized program of interventions was offered, based on the results of SUNFRAIL and the secondary scales.

Another interesting experience is the one designed and implemented by Federico II University and Hospital, which proposes SUNFRAIL+ screening for frailty in older adults, and the implementation of prevention and health promotion programs and telemonitoring. Following a specialist visit, the patient is enrolled in an adapted physical activity program, in person or on a mobile app, and participates in health promotion and psychological well-being activities, during bi-weekly meetings on nutrition, mindfulness, and fall prevention (47).

These findings contribute valuable insights to the growing body of research on frailty prevention and management, potentially informing public health policies and interventions aimed at improving the well-being of older adults. The study’s results underscore the importance of adopting a proactive approach to healthy aging, emphasizing the need for early detection and intervention strategies to mitigate the impact of frailty on older populations.

### 4.1 Limitations

The research has some limitations, such as that the prevalence obtained should not be considered representative of the entire population due to the non-random selection of the sample. Furthermore, the various Italian centers recruited the sample and administered the scales in very different and independent ways. Finally, it would be desirable for this research to be followed by a prospective long-term study that could evaluate the effectiveness of the proposed interventions on frailty, to provide evidence to support the proposal of a frailty screening of older people by the Italian National Health Service.

## 5 Conclusion

This comprehensive study provides a wealth of information on frailty assessment and its implications for older adults’ health and well-being. The findings emphasize the need for multidimensional approaches to frailty prevention and management, considering not only physical health but also psychological, social, and economic factors. As populations continue to age worldwide, research like the SUNFRAIL+ study will be instrumental in developing effective strategies to promote healthy aging and improve the quality of life for older adults.

## Data Availability

The raw data supporting the conclusions of this article will be made available by the authors, without undue reservation.
